# Greek Guidelines for the Management of COPD, a Proposal of a Holistic Approach Based on the needs of the Greek Community

**DOI:** 10.3390/jpm12121997

**Published:** 2022-12-02

**Authors:** Nikolaos Tzanakis, Epameinontas Kosmas, Andriana I. Papaioannou, Georgios Hillas, Eleftherios Zervas, Stelios Loukides, Petros Bakakos, Paraskevi Katsaounou, Afroditi Boutou, Photis Perlikos, Nikolleta Rovina, Katerina Dimakou, Paschalis Steiropoulos, Grigorios Stratakos, Philipos Emmanouil, Stavros Tryfon, Nikolaos Koulouris

**Affiliations:** 1 Department of Thoracic Medicine, University Hospital of Heraklion, Medical School, University of Crete, 71303 Heraklion, Greece; 2 Department of Pulmonary Medicine PNOH, Metropolitan Hospital, Neo Faliro, 18547 Athens, Greece; 3 2nd Respiratory Medicine Department, “Attikon” University Hospital, 15772 Athens, Greece; 4 5th Pulmonary Department, “Sotiria” Chest Diseases Hospital, 15772 Athens, Greece; 5 7th Pulmonary Department, “Sotiria” Chest Diseases Hospital, 15772 Athens, Greece; 6 1st University Department of Respiratory Medicine, National and Kapodistrian University of Athens, 15772 Athens, Greece; 7 Department of Respiratory Medicine, Evangelismos General Hospital, 15772 Athens, Greece; 8 Department of Respiratory Medicine, G. Papanikolaou Hospital, 54642 Τhessaloniki, Greece; 9 Department of Respiratory Medicine, Medical School, Democritus University of Thrace, 68100 Alexandroupolis, Greece

**Keywords:** COPD, guidelines, management, Greece

## Abstract

Despite that COPD remains one of the most common respiratory diseases worldwide, it can be managed effectively with certain treatments and, more importantly, be prevented by the early implementation of various measures. The pathology and pathophysiology of this disease continue to be studied, with new pharmacological and invasive therapies emerging. In this consensus paper, the Working Group of the Hellenic Thoracic Society aimed to consolidate the up-to-date information and new advances in the treatment of COPD. Local and international data on its prevalence are presented, with revised strategies on the diagnostic approach and the evaluation of risk assessment and disease severity classification. Emphasis is placed on the management and therapy of patients with COPD, covering both common principles, specialized modalities, and algorithms to distinguish between home care and the need for hospitalization. Although pharmacological treatment is commonly recognized in COPD, an integrative approach of pulmonary rehabilitation, physical activity, patient education, and self-assessment should be encountered for a comprehensive treatment, prevention of exacerbations, and increased quality of life in patients.

## 1. Introduction

COPD affects ~10.1% (±4.8) of the population worldwide with men and women comprising 11.8 ± 7.9% and 8.5 ± 5.8%, respectively [1]. In Greece, the prevalence of COPD has been estimated at 8.4–10.6%, with a higher impact on older patients and residents of rural areas [2,3]. The total annual healthcare financial burden of COPD in Greece has been calculated at EUR 4729/patient, with EUR 2809 being attributed to direct healthcare cost [4]. The cost of the treatment of stable COPD is EUR 1034, with inhaled drugs accounting for 71% [5].

Within the last two decades, advancements in the knowledge and understanding of COPD have broadened the management of the patients, not only depending on clinical symptoms, spirometry, and the risk of exacerbations, but also on patient-reported outcomes, such as the quality of life (QoL) and well-being of patients with COPD [6,7]. Several national and international guidelines provide physicians the most recent recommendations based on recent evidence for the diagnosis and management of the disease [7,8,9,10,11]. 

Former studies on the prevalence of COPD in Greece have indicated rather low rates when compared to the global percentage of 11.7 in 2010, thus suggesting underrecognition and underdiagnosis of COPD in real-world settings [7,12]. Moreover, it has been noted that the adherence to the previous GOLD recommendations in COPD treatment is strikingly low among clinicians in Greece, especially among general practitioners (GPs) [12,13]. Some barriers against standardized treatment of COPD are access to diagnostic procedures, a lack of recent national guidelines, and a lack of collaboration with pulmonologists [13]. In Greece, COPD patients are typically managed by specialized pulmonologists, with GPs and Internists also contributing to the diagnosis and treatment, especially in rural areas.

## 2. Methodology

Considering the unmet clinical need for the diagnosis and treatment of COPD in Greece, the Hellenic Thoracic Society has created a Working Group of renowned pneumologists in the field of COPD to elaborate on the latest updates of the international and national guidelines on COPD and to establish a practical and optimal algorithm for the diagnosis and management of COPD in busy everyday practice settings in Greece. The present work constitutes the official statement of the Hellenic Thoracic Society. The work was performed by three teams of experts in specific fields, each of which performed the literature review and made their recommendations. All members of each team needed to agree on the recommendations, while all experts reviewed the content of this work and agreed upon it.

The recommendations of the Hellenic Thoracic Society are in alignment with the overall guidelines issued by GOLD 2022 and other national societies, having incorporated the local practice and the characteristics of patients. 

## 3. Risk Factors

In most developed countries, almost 90% of COPD cases are attributable to active cigarette smoking, although 25–31.6% of participants in COPD clinical trials are non-smokers [3,14]. Other risk factors that increase COPD risk have an independent, but cumulative effect starting from intrauterine life and throughout childhood and adulthood and include the effect on the development and maturation of the respiratory system, i.e., exposure to maternal smoking and frequent childhood respiratory infections. Harmful environmental substances (outdoor and indoor air pollution) in urban and industrial areas, occupational exposure to toxic substances, such as in mine workers, farmers, and food-industry workers, and in public spaces where smokers gather, e.g., bars and restaurants (passive smoking) are some additional risk factors [15]. In patients belonging to certain vulnerable groups, exposure to pollutants from biomass burning (fireplaces, wood-burning stoves) requires particular attention for diagnostic and preventive purposes. In the case of non-smokers with typical clinical presentation and spirometry findings, secondary factors should be explored. There is evidence of heredity since children of COPD patients show a higher prevalence of the disease compared to the general population [16]. Alpha-1 antitrypsin deficiency is the best-studied genetic risk factor and should be explored in all COPD patients [17]. Female gender and low socioeconomic status are factors of increased vulnerability to smoking [18]. 

## 4. Diagnosis

COPD should be suspected in any patient >40 years old presenting with typical symptoms, who has been exposed to risk factors (predominantly smoking), irrespective of the history of recurrent lower respiratory tract infections. Often, the disease is diagnosed during a hospital admission due to an exacerbation. However, the diagnosis should always be based on technically correct post-bronchodilation spirometry. Typical respiratory symptoms of COPD include:

1. Dyspnea (progressively deteriorating, persisting, worsening with fatigue);
2. Chronic cough;
3. Increased sputum production.

Dyspnea is the most constant symptom (almost always present, particularly in advanced disease). Cough and sputum production are present in 50% of cases. In early disease, sputum production and dyspnea may not be perceived by the patient due to their gradual appearance. These symptoms are usually accompanied by one of the following:

1. History of exposure to smoking (commonly > 10 pack-years);
2. History of exposure to environmental or occupational air pollutants, smoke, dust, and chemicals;
3. Frequent respiratory tract infections;
4. Family history of COPD.

Clinical examination, though an integral part of patient assessment, is not a helpful diagnostic tool. The typical respiratory sounds heard on auscultation may not be identifiable, especially in early COPD.

Symptoms of weakness, decreased appetite, and weight loss are indicative of severe COPD, but may suggest the presence of a concomitant disease (heart failure, cancer, tuberculosis). Swelling of the lower extremities may be the sole manifestation of pulmonary hypertension and right heart failure, mostly present in advanced stages. The coexistence with heart failure is seen mostly in elderly patients and should be suspected when symptoms’ severity (especially dyspnea) is disproportionate to the spirometric results. Symptoms of anxiety and depression are common and worsen disease prognosis through exacerbations (Table 1).

## 5. Diagnostic Tests

### 5.1. Spirometry

A COPD diagnosis is confirmed when irreversible airflow obstruction is seen in post-bronchodilator spirometry, i.e., FEV_1_/FVC index ≤ 0.70 or 70%. However, the threshold of 70% may cause a more frequent diagnosis of COPD in the elderly, whereas a less frequent diagnosis in adults <45 years of age. While the fixed ratio of FEV_1_/FVC < 70% is recommended in some European countries and Russia, the guidelines from the Czech Republic, Italy, Poland, and Sweden suggest that the post-bronchodilator FEV1/FVC ratio is less than the lower limit of normal (LLN) in COPD patients [8,10]. In the Spanish guideline, it is recommended to use the fixed ratio in patients <50 years and >70 years of age for whom LLN is recommended [10]. GOLD 2022 recommends the use of the FEV_1_/FVCratio over the FEV_1_/VCratio to avoid lower values, especially in patients with a significant airflow limitation [19]. 

The hot point of the discussion about the evaluation of lung function is the selection of the most suitable tool for different age groups. While post-bronchodilator FEV_1_/FVC may be false negative in patients <50 years or with borderline values, it may be an unreliable parameter in patients >80 years since it is greatly affected by ageing [12]. Therefore, although the limit of 0.70 is an accurate threshold for patients aged between 50 and 80 years, LLN may be a more reliable and accurate parameter for the age groups of <50 years and >80 years. 

A practical algorithm proposed for the calculation of LLN values involves subtracting 0.10 or 10% from the patient’s predicted FEV_1_/FVC ratio and using the resulting value as the corresponding LLN with a clinically acceptable degree of accuracy [20,21]. The spirometry-based diagnosis of COPD should be assessed and questioned when the severity of dyspnea is disproportionate to spirometric measurements. The FEV_1_%pred value is not considered for the diagnosis, but it is essential for the classification of severity. However, a change in post-bronchodilator FEV_1_ of >400 mL warrants assessment for coexistent asthma or may be a case of asthma only in a smoker [22].

### 5.2. Other Tests and Examinations

More detailed lung function tests are helpful in differential diagnosis. Lung volumes, perfusion, and arterial blood gases (ABGs), though in some cases supportive, are not necessary for diagnosis. It is, however, extremely important in assessing disease severity. Cardiopulmonary Exercise Testing is a dynamic, non-invasive method helpful in differential diagnosis and in assessing possible extrapulmonary causes of dyspnea. A chest X-ray and CT scan, though not helpful in the COPD diagnosis, may be performed. These imaging tests aid in the identification and assessment of common comorbidities, especially in the presence of other signs or symptoms. Echocardiographic assessment of the heart, hematologic and biochemical tests, cytology, and sputum culture are used to fully investigate comorbidities or alternative diagnoses.

## 6. Classification of Severity and Assessment of Risk

Classifications of severity and risk level will define the prognosis and the extent of stable treatment that is required. Staging is determined by quantitative assessment of airway obstruction damage based on the percentage of FEV_1_ decrease from the predicted value, being consistent with the symptom severity, exacerbation frequency, and number/severity of comorbidities (Table 2).

Symptoms can be quantified using tools of symptom severity and quality of life (QoL), such as the St. George’s Respiratory Questionnaire, the COPD Assessment Test, the Clinical COPD Questionnaire, and the modified Medical Research Council Dyspnea Scale. 

## 7. Special Diagnostic Considerations 

### 7.1. Diagnostic Difficulty 

Since symptoms are non-specific and chronic, patients may integrate them into their daily lives and fail to assess or report them.

In some cases, spirometry might be non-diagnostic. Underdiagnosis (most often) or overdiagnosis of COPD exists in the following cases [23]:

1. In borderline FEV_1_/FVC or LLN.
2. In technical inability to perform spirometry, i.e., tracheostomy.
3. In concomitant diseases that may influence critical spirometric values, e.g., congestive heart disease or kyphoscoliosis.

### 7.2. Coexistence of Asthma and COPD 

Asthma and COPD are two independent disease entities with different clinical and pathophysiological characteristics [22,24,25]. However, their coexistence can be suspected when common clinical characteristics of both diseases are present. It is difficult to assess the prevalence of such cases, though this ranges in the literature between 15 and 45% [22]. Patients with this specific phenotype present with a higher disease severity compared to typical COPD and number of comorbidities and worse disease control despite maximum treatment [26,27,28]. Though eosinophilic or T2-high are the dominant types of asthma, a group of patients present with neutrophil-dominated (T2-low) inflammation [29]. Several reports suggest the presence of eosinophilic inflammation in severe COPD exacerbations and a subset of patients with stable disease [30,31]. Moreover, irreversible airway obstruction in asthma is often seen, especially in patients with severe asthma and in asthmatic smokers [32], while a response of >12% to bronchodilation in COPD is reported in ≥45% [33]. We propose a clinical algorithm for the identification of asthma–COPD overlap syndrome (Online Resource 1), being aware that it is based on empirical and clinical considerations; its usefulness remains to be assessed in prospective studies [34].

Online Resource 1: Diagnostic approach to the asthma–COPD overlap phenotype. Footnotes: COPD, chronic obstructive pulmonary disease; NO: FeNO fraction of exhaled nitric oxide; Eos, eosinophils; SPT: skin prick test.

## 8. Classification of Disease Outcome Risk

**Figure 1:**
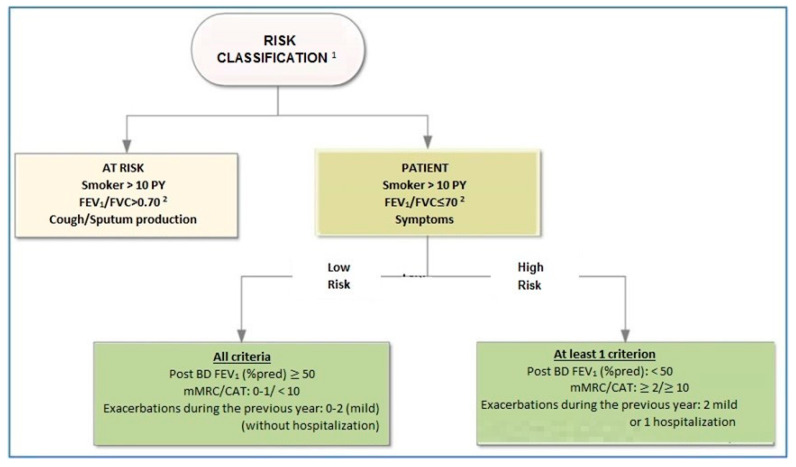
Steps in the treatment of COPD. Abbreviation: COPD, chronic obstructive pulmonary disease. ^1^ Evaluate comorbidities carefully. ^2^ Use the Lower Limit of Norma, (LLN) in borderline values especially in patients bellow 45 and over 80 years of age.

We propose a two-level risk classification (Figure 1). The group of individuals at risk for developing COPD is presented to promote medical awareness and counselling for the reduction of modifiable risk factors.

The parameters included in this classification have been shown to be of prognostic value for mortality [35].

## 9. Treatment of COPD

**Figure 2:**
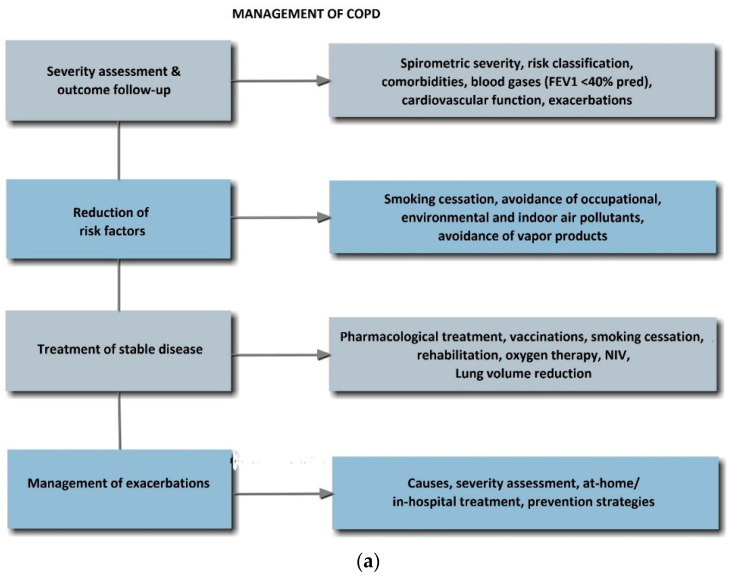

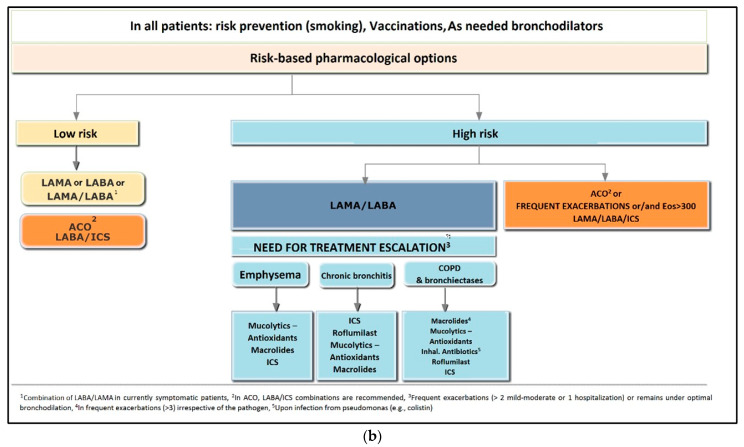
(**a**) Classification of prognostic risk. Footnote: 1 Carefully evaluate comorbidities. 2 Use LLNs in patients with borderline values, especially in patients <45 and >80 years of age. Abbreviation: LLN, lower limit of normal. (**b**) Treatment algorithm for stable COPD. Abbreviation: COPD, chronic pulmonary disease.

The effective treatment of COPD includes four main steps (Figure 2a).

A successful COPD treatment has the following objectives:

- Decelerate disease progression;
- Symptom control;
- Improve exercise tolerance;
- Improve QoL;
- Prevent disease complications;
- Prevent/treat exacerbations;
- Reduce mortality.

### 9.1. Treatment of Stable COPD

Treatment of stable disease aims at reducing symptoms and lowering the frequency and severity of exacerbations. General therapeutic measures include smoking cessation, an increase in physical activity, a proper diet, management of comorbidities, and vaccination. Pharmacological treatment is escalated based on the risk classification (Figure 2b).

### 9.2. Pharmacological Treatment of Low-Risk Patients

The treatment of low-risk patients is based on long-acting beta2-agonists (LABAs: vilanterol, indacaterol, olodaterol, salmeterol, formoterol) and long-acting muscarinic antagonists (LAMAs: aclidinium, glycopyrronium, umeclidinium, tiotropium). These medications improve patient symptoms and lung function and reduce exacerbations [36,37,38,39]. It is recommended that LAMAs be initiated first as they seem to be more effective in reducing exacerbations. In some cases, a LAMA/LABA combination should be administered to low-risk patients with prominent symptoms or unsatisfactory response to monotherapy [40]. Anti-inflammatory therapy with inhaled corticosteroids (ICSs) is not required in low-risk patients, except for those with established coexisting asthma, in whom the initiation of the LABA/ICS combination is recommended.

### 9.3. Treatment of High-Risk Patients

A dual bronchodilator is indicated in high-risk patients. If the patient remains symptomatic or continues to experience exacerbations (>2 mild or 1 requiring hospitalization), treatment escalation is required based on the patient’s phenotype. In chronic bronchitis, the addition of ICS on LABA/LAMA is the first choice for the reduction of exacerbation frequency [41]. 

Another option in patients with chronic bronchitis and FEV_1_ < 50% is the phosphodiesterase-4 inhibitor roflumilast [42]. 

Mucolytics such as erdosteine, carbocysteine, and N-acetylcysteine have an excellent tolerance and safety profile, with studies showing a reduction in exacerbations regardless of phenotype [43]. 

Macrolides (mostly azithromycin) lead to the reduction of exacerbations in severe COPD. All existing studies had a maximum duration of 1 year, therefore not allowing reliable recommendations for treatment extension beyond this period, and used 250 or 500 mg of azithromycin daily for 3 days/week. The development of bacterial resistance, hepatotoxicity, and hearing loss are potential problems [44]. 

Inhaled antibiotics (colimycin) can be used in the mixed COPD–bronchiectasis phenotype with *Pseudomonas* colonization with specialized therapy for both diseases needed [45]. In high-risk patients with coexisting asthma, initial triple therapy with LAMAs/LABAs/ICS is recommended. ICSs may be added for patients with emphysema not controlled by the dual bronchodilator therapy, though their efficacy is expected to be lower than those with chronic bronchitis. For patients who are classified as high-risk due to frequent exacerbations and presenting with an eosinophil blood count of >300 cells/μL, an initial triple therapy with LAMAs/LABAs/ICS is also recommended [46,47,48,49].

## 10. Vaccinations

Vaccination is essential in COPD. Patients should be vaccinated against SARS-CoV-2, *influenza*, and *pneumococcus* regardless of their age. *Influenza* vaccination is one of the safest and most effective preventive measures, as it can prevent up to 60% of adult cases and reduce the number of COPD exacerbations and mortality [50,51,52,53]. Despite ongoing efforts, only about 50% of COPD patients have been vaccinated in the past few years [54]. 

## 11. Precautions

*Influenza* immunization should be provided to healthcare professionals who are in direct contact with COPD patients:

(A) To protect themselves and their patients;
(B) To limit the transmission of *influenza* in healthcare and social care facilities;
(C) To protect individuals who may have had a reduced immune response to their vaccinations.

Pneumococcal vaccination is recommended with the 23-valent polysaccharide vaccine (PPSV23) or the 13-valent pneumococcal conjugate vaccine (PCV13) (Online Resources 2 and 3). *Pneumococcus* is the most common cause of lower respiratory tract infections, with its incidence in COPD being almost double [55]. 

Online Resource 2: Guidelines for the administration of pneumococcal vaccines to previously unvaccinated adult patients with chronic obstructive pulmonary disease. Online Resource 3: Guidelines for the administration of pneumococcal vaccines to previously vaccinated adult patients with chronic obstructive pulmonary disease (the star symbol next to the vaccine indicates the vaccine has already been administered).

Based on the antigenicity of each vaccine, a sequence has been established to achieve the best-possible immunogenicity [56]. The grouping of COPD patients is determined based on (a) the age limit of 65 years and (b) whether the patient has previously received one of the two vaccines. 

The concomitant administration of ICSs in the standard moderate doses used in COPD and short-term regimens of systemic corticosteroids (up to 30 mg prednisolone for 5 days) have no effect on the post-vaccination immune response.

The PPSV23 vaccine may be co-administered with the seasonal inactivated trivalent *influenza* vaccine (TIV) and the seasonal inactivated quadrivalent *influenza* vaccine (QIV). The PCV13 vaccine may be co-administered with the TIV vaccine, but a combination with the QIV vaccine, though not contraindicated, may diminish the PCV13-produced immune response [57].

## 12. Smoking Cessation in COPD

Smoking cessation is the most effective intervention for slowing down the course of COPD [25]. Nicotine dependence is a chronic condition (ICD-10-CM/F17) [58] for which safe and effective treatment options are available. A combination of behavioral and pharmacological treatment produces excellent results and should be adopted by physicians in their daily practice. If effective resources and time are dedicated to smoking cessation, success rates of 25% can be achieved in the first year [59].

### Pharmacological Treatment for Smoking Cessation

There are three medications for smoking cessation, used alone or in combination, with comparable efficacy rates:

- Nicotine replacement therapy is available in various forms (patches, sublingual tablets, gums, spray, inhaler) and dosing regimens.
- Varenicline.
- Bupropion.

The recommended treatment duration is three months and may be extended to six months in highly addicted smokers. E-cigarettes of any kind (vaping, IQOS, etc.) are not recommended by the European Respiratory Society and the Hellenic Thoracic Society as a smoking cessation method [60].

## 13. Pulmonary Rehabilitation

Pulmonary rehabilitation is a multifaceted and comprehensive intervention for patients with COPD, based on a detailed assessment of the patient’s status and characterized by a personalized treatment approach, which includes a workout schedule, individual training, behavioral therapy, and psychological support. It aims to improve the patient’s physical and psychological status and to enhance long-term compliance to health-promoting behaviors, while it can be performed at home [61,62].

## 14. Diagnosis and Treatment of COPD Exacerbation

### 14.1. Definition

A COPD exacerbation is defined as a recent worsening of the patient’s symptoms (cough, dyspnea, sputum production) and/or as the onset of new symptoms requiring a change in the patient’s regular treatment.

### 14.2. Classification of COPD Exacerbations

Depending on their severity, exacerbations are classified as follows:

A. Mild: Exacerbations requiring an increase in the use of bronchodilators for <2 days outside the hospital.
B. Moderate: Exacerbations requiring administration of antibiotics with or without oral corticosteroids.
C. Severe: Exacerbations requiring hospitalization.

There are exacerbations not reported by patients to healthcare professionals that also play an important role in the patient’s general health status. Patients should be trained to recognize exacerbation symptoms and seek medical care, while physicians should keep in mind that the number of exacerbations is often underestimated [36,63,64,65,66].

### 14.3. Assessment of COPD Exacerbation

The initial assessment of an exacerbation will determine whether a patient needs to be managed at home or be hospitalized. Patient’s information that needs to be evaluated during the initial assessment are:

- Symptoms severity (dyspnea, confusion, etc.).
- Clinical signs of COPD and/or comorbidities.
- Oxygen saturation.
- Comorbidities (cardiovascular diseases, diabetes mellitus, chronic kidney disease, etc.).
- Respiratory function and symptoms (cough, dyspnea, etc.) compared to the patient’s stable state.
- History of previous exacerbations and their treatment.
- History of hospitalizations for exacerbations.
- Patient’s current stable treatment.

### 14.4. Cause of Exacerbations

Infections are the main cause of exacerbations. Infectious exacerbations are caused by bacteria (40–60%), mainly *Haemophilus influenzae, Moraxella catarrhalis,* and *Streptococcus pneumoniae* or virus (30–40%), mainly *Rhinovirus*, *RSV*, and *Influenza* viruses. In special situations, e.g., frequent exacerbations with hospitalization in patients with severe airway obstruction, Gram-negative bacteria may prevail, with *Pseudomonas aeruginosa* being the main representative.

Non-infectious exacerbations include those related to exposure to pollutants and those of unknown etiology (conditions either representing independent diseases or diseases coexisting with the main cause of the exacerbation, such as an infection). Typical examples are congestive heart failure, pulmonary embolism, and pneumonia. 

The clinical criteria of an infectious exacerbation and the indication for antibiotic administration are those introduced by Anthonisen et al. [67].

### 14.5. Indications for Referral to the Hospital

A referral to the hospital is necessary when any of the following is observed [64,65,68]:

1. Serious symptom deterioration: sudden development of resting dyspnea, tachypnoea, confusion, etc.
2. Appearance of new physical signs (e.g., cyanosis, peripheral edema).
3. Oxygen desaturation with SaO_2_ < 90% in patients not previously on oxygen therapy at home or worsening of pre-existing respiratory failure.
4. Presence of serious comorbidities (e.g., congestive heart failure, chronic kidney disease).
5. Inability to identify the cause of the exacerbation.
6. Failure of an exacerbation to respond to initial management.
7. Insufficient home care.

### 14.6. Indications for Hospitalization

Upon assessment of an exacerbation in the emergency department (ED), a hospitalization is deemed necessary when any of the following is observed [64,65,68]:

1. Presence of severe signs/symptoms (resting dyspnea, tachypnoea, labored breathing, confusion), despite initial management of the exacerbation in the ED.
2. Persistent respiratory failure requiring high oxygen mixtures and/or severe/worsening respiratory acidosis requiring non-invasive mechanical ventilation (NIV).
3. Hemodynamic instability.
4. Appearance of new physical signs (e.g., cyanosis, peripheral edema).
5. Presence of serious comorbidities (e.g., heart failure, arrhythmia).
6. Insufficient home care.

### 14.7. Indications for HDU/ICU Admission

A patient experiencing an exacerbation should be transferred to a high dependency unit (HDU) or intensive care unit (ICU) when any of the following is observed [64,65,68]:

1. Persistent or worsening respiratory failure and/or severe/worsening respiratory acidosis (pH < 7.35), despite oxygen therapy and NIV.
2. Change in mental status: confusion, coma.
3. Need for invasive mechanical ventilation.
4. Hemodynamic instability, requirement of vasoconstrictors.

### 14.8. At-Home and In-Hospital Treatment of an Exacerbation

**Figure 3:**
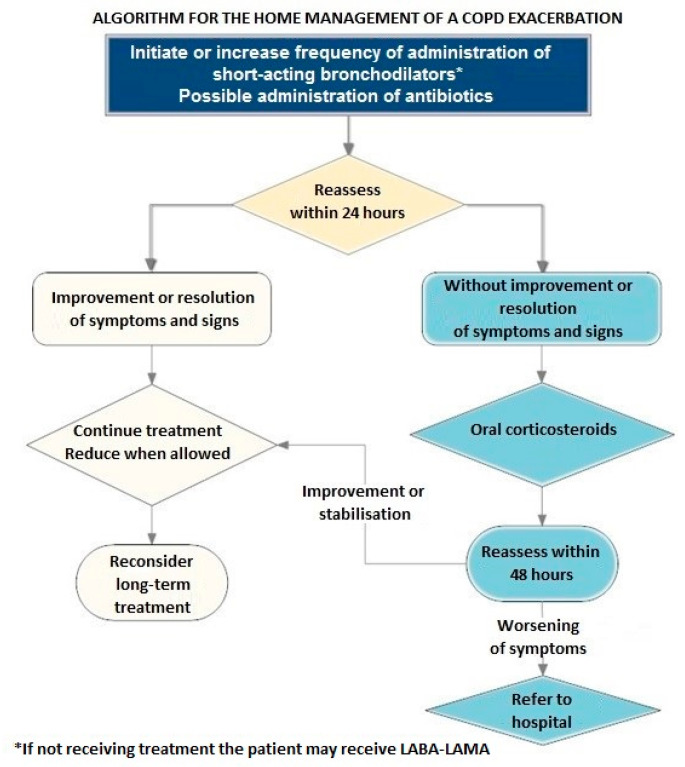
Home management of a COPD exacerbation. Abbreviation: COPD, chronic pulmonary disease.

The treatment goal in a patient with an exacerbation is to limit its negative effects and avoid subsequent exacerbations. The algorithm for home management of an exacerbation is shown in Figure 3.

### 14.9. Steps in the Treatment of a COPD Exacerbation 

#### 14.9.1. A: Antibiotics

The administration of antibiotics reduces mortality and treatment failures. It is recommended that patients with Anthonisen type I and type II exacerbations, those with severe exacerbation requiring hospitalization, and/or those with exacerbations requiring mechanical ventilation (invasive or non-invasive) should receive antibiotic treatment.

The decision to administer antibiotics during exacerbations should be based on the use of inflammatory biomarkers, although the data remain controversial. CRP does not appear to be a reliable biomarker, as its levels increase in both bacterial and viral exacerbations. By contrast, the use of procalcitonin appears to provide the ability to identify patients requiring treatment with antibiotics, thus minimizing their unnecessary use [69].

Recommended antibiotics are aminopenicillins with clavulanic acid, macrolides, and tetracyclines. In frequent exacerbations, severe COPD, use of mechanical ventilation, or frequent antibiotic use, coverage for Gram-negative pathogens is also required. Treatment should always be adjusted based on cultural results or the history of colonization.

#### 14.9.2. B: Bronchodilators 

Short-acting beta2-agonists and/or short-acting anticholinergics are suggested at regular intervals to ensure the patient’s relief from dyspnea. Initially, an hourly administration for the first 2–3 h and then every 2–4 h depending on the patient’s needs is recommended. They may be administered with pressurized metered-dose inhalers (with or without spacers) or with nebulizers (useful in patients with more severe dyspnea) [70]. LABAs and LAMAs that comprised part of the patient’s stable treatment do not need to be interrupted, and if so, they should be re-administered as soon as possible and definitely before the patient’s discharge from the hospital.

#### 14.9.3. C: Glucocorticosteroids

Glucocorticosteroids shorten hospitalization length, improve FEV_1_ and oxygenation, and reduce the risk of relapse [71], but do not seem to affect mortality. Caution must be exercised in patients at high risk for adverse effects, such as those with diabetes mellitus, arterial hypertension, bronchiectasis, osteoporosis, immunosuppression, etc. The recommended dose is 30–40 mg/day of prednisone or equivalent for 5 days [72]; no tapering is required. In patients treated with systemic steroids over the past month (due to an exacerbation), re-administration is not required. The administration of slow-release corticosteroids should be avoided.

#### 14.9.4. D: Other Supportive Measures 

Full patient support should be provided based on the severity of the exacerbation.

- Both at home or in the hospital, early mobilization and sufficient management and treatment of concomitant diseases should be addressed.
- In hospitalized patients, the following should be considered:
  1. In those with respiratory failure, oxygen therapy should be administered, and ABG should be regularly monitored.
  2. Careful balance of fluids.
  3. Low-molecular-weight heparin should be administered in prophylactic doses (excluding patients treated with anticoagulants for other reasons, e.g., atrial fibrillation who should receive a full dose).
  4. Finally, the differential diagnosis and recognition of potential concomitant diseases that mimic exacerbation (e.g., pulmonary embolism, pneumothorax, pneumonia, etc.) are required.

#### 14.9.5. E: Patient Education

Patients should receive education regarding the disease’s nature and severity, exacerbation recognition and early pursue of medical care, and the importance of pharmacological and non-pharmacological interventions and preventive measures to avoid exacerbations [73].

#### 14.9.6. Additional Management

Exacerbations accompanied by respiratory failure require hospitalization and supplemental oxygen and/or mechanical ventilation (invasive or non-invasive).

#### 14.9.7. Oxygen Therapy

Oxygen therapy should be administered to any patient with respiratory failure with the goal of a SpO_2_ of 88–92%. During an exacerbation, the use of controlled oxygen delivery devices (Venturi masks) is generally preferred over the use of nasal cannulas, as they allow a more accurate estimation of the provided oxygen ratio. Frequent blood gas measurement is required to monitor PaCO_2_ and pH levels, due to the risk of developing respiratory acidosis or worsening of pre-existing respiratory acidosis [74].

#### 14.9.8. Non-Invasive Mechanical Ventilation

NIV should be the initial mode of mechanical ventilation applied to any patient with an exacerbation that has no absolute contraindications, as it improves gas exchange, reduces the work of breathing and the need for intubation, shortens the length of hospital stay, and improves survival [75]

#### 14.9.9. Indications for NIV 

At least one of the following indicates the need for NIV:

- Respiratory acidosis (PCO_2_ > 45 mmHg and arterial blood pH ≤ 7.35).
- Severe dyspnea with clinical signs suggestive of respiratory muscle fatigue, increased work of breathing, or both, such as the use of respiratory accessory muscles, paradoxical motion of the abdomen, or retraction of the intercostal spaces.
- Persistent hypoxemia despite high-concentration oxygen therapy.
- Ιn cases in which NIV fails or is contraindicated, intubation and invasive ventilation should take place.

#### 14.9.10. High-Flow Nasal Canula 

HFNC delivers heated and humified oxygen via special devices in rates up to 60 L/min in adults. It has several physiologic benefits resulting in a decrease in the work of breathing, improvement of gas exchange, and dynamic compliance and transpulmonary pressures. During COPD exacerbations, HFNC has been shown to be non-inferior compared to NIV in decreasing PaCO_2_, although approximately 1/3 of patients might finally require NIV [76]. The use of HFNC during COPD exacerbations is recommended to be used only after a trial of NIV in COPD patients with hypercapnic acute respiratory failure [77].

#### 14.9.11. Laboratory Testing and Imaging

Laboratory testing and the ability to perform imaging techniques apply to any exacerbation, whether managed at home or in the ED, with or without hospitalization. 

In patients treated at home (when required):

1. Complete blood count;
2. Biochemistry;
3. Chest X-ray;
4. Quantitative CRP;

In patients visiting the ED or requiring hospitalization:

5. Complete blood count;
6. Biochemistry;
7. Quantitative CRP;
8. Procalcitonin
9. D-dimers (only when pulmonary embolism is suspected);
10. Troponin and NT-pro BNP (if the deterioration of congestive heart failure and/or suspicion of a coronary event must be assessed)
11. ECG;
12. Echocardiogram, if indicated;
13. Sputum culture for common pathogens before the initiation of antibiotics;
14. Chest X-ray (F&P)
15. Chest CT or other imaging modality, e.g., CTPA, only if indicated.

It is important to assess whether the at-home management of an exacerbation may not require any laboratory or imaging tests and may be evaluated solely on clinical markers. However, there are also approaches concerning the investigation of complications of comorbidities, mainly cardiovascular events. As such, tests including cardiac biomarkers and/or echocardiography are required. When pulmonary embolism is highly suspected, D-dimer measurement and CT pulmonary angiography should be performed [78]. There are insufficient data supporting the role of peripheral eosinophilia as a factor in therapeutic decision-making during an exacerbation [79]. 

## 15. Oxygen Therapy at Home (Long-Term Oxygen Therapy)

LTOT at home is defined as the continuous oxygen delivery at a flow capable of achieving SpO_2_ > 90% with a duration of ≥15 h/day. LTOT has been demonstrated to increase survival in chronic respiratory failure with severe resting hypoxemia [80,81]. 

In patients with mild hypoxemia without respiratory failure, though alleviating dyspnea, LTOT does not seem to increase survival [80,81]:

- Indications for LTOT: Presence of significant resting hypoxemia with pO_2_ < 55 mmHg or with 55 mmHg < pO_2_ < 60 mmHg and concomitant heart failure, polycythemia, and/or pulmonary hypertension [80,81].
- Minimum duration of LTOT should be 15 h/day, while continuous oxygen therapy (24 h/day) is possible. Daytime hypercapnia is not a contraindication for LTOT [80,82,83].
- In patients with hypoxemia during sleep, LTOT is not currently recommended, as it does not seem to improve mortality [84].
- Patients with hypoxemia during physical activity may benefit from the use of portable oxygen delivery during activity if they qualify for LTOT at rest. Portable oxygen delivery in these patients appears to increase compliance and works towards achieving the target of ≥15 h/day.
- Patients with severe hypoxemia only during physical activity without resting hypoxemia do not require LTOT [85,86].
- Following the initiation of LTOT, a follow-up assessment of each patient after 4–6 weeks is recommended, to evaluate the need to continue oxygen [87,88,89].
- Oxygen delivery flow is titrated as follows:
- Initial flow of 1 L/min and gradual increase by 1 L/min every 20 min until SpO_2_~90% is achieved. The initial check is performed via a pulse oximeter, and the final flow is titrated based on ABG [90,91].
- Initiation of LTOT and flow titration in chronic hypercapnic patients should be performed under close monitoring and with measurements of ABG performed after any change in the oxygen flow, including final titration [83].
- For patients who continue to smoke, LTOT should be administered upon discussion with the patient and following potential risk/benefit assessment [92,93].

## 16. Non-Invasive Ventilation in Stable COPD

The use of NIV in patients with hypercapnic respiratory failure has been shown to improve their symptoms, ability to exercise, QoL, survival, and functional disease parameters [94,95,96,97,98]. Discontinuation of NIV appears to reverse its beneficial effects [99]. Studies conducted in the previous decade did not manage to show that NIV increases survival [95,100,101].

Patients with COPD suffer from more intense symptoms during the night, due to the normal changes during sleep (changes in cortex stimuli, respiratory center sensitivity, chemoreceptor sensitivity, and lung mechanics). These changes result in hypoventilation in comparison to wakefulness and in subsequent hypoxemia and hypercapnia, which are much more severe in patients with established hypercapnic respiratory failure.

Alongside the standard strategy of low-intensity NIV, that of high-intensity NIV has been developed. A combination of high pressures and an endogenous rate of mandatory ventilation is used with the goal of reversing hypercapnia and achieving normocapnia, if possible [102]. The application of this strategy has demonstrated improvement in a number of parameters in patients with stable COPD and hypercapnic respiratory failure, including lung function tests, the ability to exercise, dyspnea, and QoL [103,104,105]. These results were also confirmed by studies comparing high-intensity NIV with the standard low-intensity approach [106]. High-intensity NIV also managed to improve survival in cases of severe hypercapnic respiratory failure [105]. Moreover, the number of hospitalizations in frequent exacerbators was reduced [107], while NIV discontinuation led to significant clinical deterioration [108,109,110,111].

The current literature recommends the use of NIV to restore normocapnia for ≥5 h/day in those already receiving the optimal treatment for the COPD and concomitant conditions, who have:

- Daytime hypercapnia with PaCO_2_ ≥ 50 mmHg (absence of acute exacerbations for ≥3 weeks).
- Nocturnal hypercapnia with PaCO_2_ ≥ 55 mmHg (absence of acute exacerbations for ≥3 weeks).

Regarding the titration of NIV settings, the following are recommended:

- Ideally, titration should be performed in a hospital setting and, in any case, under strict medical supervision.
- Initial titration for daily use.
- Initially low EPAP (3–4 cmH_2_O) and IPAP (14–16 cmH_2_O) with a low backup respiratory rate.
- Gradual IPAP increase to the maximum tolerated level (usually ~30 cmH_2_O, but this value may differ from patient to patient; range: 20–40 cmH_2_O).
- Respiratory rate should increase up to the patient’s stable respiratory rate.
- Perform a slight increase of EPAP at 4–6 cmH_2_O, to avoid hyperinflation.
- Higher EPAP values are necessary in cases of concomitant obstructive sleep apnea (OSA).
- The application of NIV at night should only be started after a period of adjustment for the patient to get used to its daytime use.
- During this period, changes in the settings may be necessary to maximize patient comfort and compliance.
- In case of poor tolerance to nighttime use or suspicion of concomitant OSA, polysomnography may be required for the titration of pressures.

## 17. Surgical and Bronchoscopic Treatment of Severe Pulmonary Emphysema

Pulmonary emphysema, which is characterized by parenchymal destruction, elastic recoil reduction, and premature airway closure, causes lung hyperinflation and air trapping. 

Surgical excision of part of the affected hyperinflated lung (lung volume reduction) creates vital space in the thoracic cavity, reduces hyperinflation, enhances the mechanical properties of the respiratory tract, and improves the patient’s dyspnea and tolerance to physical activity [112]. 

There are two main approaches for achieving reduction/upper lobe atelectasis depending on the clinical, radiological, and functional characteristics of patients (Online Resource 4):

A. Reversible airway obstruction techniques leading to target segments (placement of one-way valves at the segment or lobe level) given that there is no collateral ventilation and the fissure between the lobes is complete.
B. Irreversible techniques directly reducing the parenchyma of the target lobe (coil placement, vapor ablation in the case of incomplete fissure and the presence of collateral ventilation. Adequate representation of lung parenchyma is required for the use of endobronchial coils.

Online Resource 4: Treatment selection algorithm.

## 18. Conclusions

In conclusion, this consensus paper covers the cornerstones of the diagnosis, management, and personalization of COPD treatment based on recent guidelines, unmet needs, and patient characteristics in Greece. This paper favors the use of LLN in patients younger than 50 years and older than 80 years over the fixed fraction FVE_1_/FVC. The Working Group also presented a clinical algorithm for the identification of asthma–COPD overlap syndrome, algorithms to distinguish patients to be treated at home versus hospital, and an algorithm for the treatment of stable COPD in Greece. We propose a treatment two-level risk classification and selection algorithm. The Working Group strongly underlines that vaccination rates for influenza and pneumococcus should be increased among COPD patients. Since smoking is the most prominent predisposing factor for COPD in Greece, encouraging patients to quit smoking should be one of the major responsibilities of treating clinicians along with the implementation of pulmonary rehabilitation. The cause and severity of exacerbations should be well documented, and appropriate pharmacological treatment should be initiated either at home or in the hospital. If required, invasive interventions should be employed for severe pulmonary emphysema to enhance the main mechanics of the respiratory system. 

Greece differs from other countries in that the main management of COPD patients is performed by general practitioners. The structure of the healthcare system in Greece leads the majority of COPD patients to be treated by specialists, which requires a better knowledge of the disease to a greater extent and the performance of spirometry at regular intervals. Furthermore, the specialists should also provide access to special, often personalized treatments and also offer a holistic approach to disease management by providing both pharmacological and non-pharmacological interventions. Due to the frequency of comorbidities in patients with COPD, the respiratory specialists in Greece must also collaborate with other specialists such as cardiologists, endocrinologists, and psychiatrists.

The structure of the healthcare system, which is, as we said, based on specialists and not on general practitioners, also leads to several unmet needs. Treatment from specialists results in increased expectations from the patients, which require better knowledge regarding disease management and increased availability for access to modern treatments or clinical trials. Furthermore, the need of the patients to participate in the self-management of their disease requires better communication and educational skills from the physician and also better skills in the use of technology to achieve better disease control and follow-up.

We believe that the Hellenic Thoracic Society’s recommendations can contribute to the development and update of guidelines on similar diseases and patient profiles in other countries, offering significant clinical experience to the global knowledge database.

## Figures and Tables

**Table 1:** Differential diagnosis of COPD.

Diagnosis	Main Characteristics
COPD	Onset always in adulthood History of exposure to risk factors (smoking) Symptoms for several years with little fluctuation over time, gradually deteriorating
Asthma	Onset usually in childhood There is often concomitant atopy, rhinitis, or eczema, family history Symptom severity varies over time; symptoms are worse at night or in the morning
Bronchiectasis	Large amount of daily sputum production LTypical X-ray/HRCT findings
Chronic heart failure	History, clinical signs of heart disease Cardiomegaly on chest X-ray Spirometry indicative of a restrictive, rather than an obstructive, syndrome
Bronchiolitis (infectious or autoimmune)	Young non-smokers Often coexists with rheumatoid arthritis or other rheumatic/autoimmune disease History of acute exposure to toxic aerosols
Tuberculosis	Bloody or purulent sputum

Abbreviations: COPD, chronic obstructive pulmonary disease; HRCT, high-resolution computed tomography.

**Table 2:** COPD stages and association with symptoms, exacerbations, and comorbidities.

Stages of Severity (FEV_1_ % Pred.)	Symptoms	Exacerbations	Comorbidities
Stage 1 (>80)	Dyspnea with moderate physical exertion, little/no effect on physical activity, cough and/or sputum production	Frequency and severity increase per stage	Observed at all stages
Stage 2 (79–50)	Increased dyspnea, e.g., after walking 100 m on level ground, decreased physical activity, cough and sputum production, recurrent respiratory tract infections		
Stage 3 (49–30)	Dyspnea with little physical exertion, daily cough and sputum production, a significant decrease in daily activity, and symptoms of frequent infections; Stage 4 patients usually have severe hypoxemia and/or respiratory failure		
Stage 4 (<30)			

Abbreviations: COPD, chronic obstructive pulmonary disease; FEV1, forced expiratory volume in 1 s.

## Data Availability

Not applicable.

## Abbreviations

ABG	Arterial blood gases
ED	Emergency department
HDU	High dependency unit
ICU	Intensive care unit
LABA	Long-acting beta2-agonists
LAMA	Long-acting muscarinic agonists
LLN	Lower limits of normal
LTOT	Long-term oxygen therapy
NIV	Non-invasive mechanical ventilation
OSA	Obstructive sleep apnea
PCV13	13-valent pneumococcal conjugate vaccine 2
PPSV23	3-valent polysaccharide vaccine
QIV	Quadrivalent influenza vaccine
QoL	Quality of life
SPT	Skin prick test
TIV	Trivalent influenza vaccine
